# Discussion on the Differences in Aroma Components in Different Fragrant Rice Varieties during Storage

**DOI:** 10.3390/life13102063

**Published:** 2023-10-15

**Authors:** Jui-Chia Lee, Kai-Min Yang, Chin-Sheng Wu, Lee-Ping Chu, Wei-Miao Jiang, Hsin-Chun Chen

**Affiliations:** 1 Hualien District Agricultural Research and Extension Station, Ministry of Agriculture, Hualien 973, Taiwan; xu3bjo4ru8@hdares.gov.tw; 2 Department of Food Science, National Quemoy University, Kinmen 892, Taiwan; a9241128@gmail.com; 3 Department of Pharmacy, China Medical University Hospital, Taichung 404, Taiwan; 092189@tool.caaumed.org.tw; 4 Department of Orthopedics, China Medical University Hospital, Taichung 404, Taiwan; chu.leeping@gmail.com; 5 Department of Cosmeceutics, China Medical University, Taichung 406, Taiwan

**Keywords:** fragrant rice, headspace solid-phase microextraction (HS-SPME), gas chromatography-mass spectrometry (GC-MS)

## Abstract

Rice (*Oryza sativa* L.) is an important food crop in Taiwan, among which fragrant rice is highly regarded for its special aroma when cooked. During the storage of fragrant rice, the aroma components will change, which will affect the aroma quality of fragrant rice. Therefore, headspace solid-phase microextraction (HS-SPME) was used in this study, combined with gas chromatography and gas chromatography-mass spectrometry, to analyze the difference in the aroma components of Taikeng No. 4 (TK4), Tainung No. 71 (TN71), Kaohsiung No. 147 (KH147), and Taichung No. 194 (TC194) fragrant rice. A total of 28 aroma components were identified in the four varieties of fragrant rice, and the main components were all Nonanal. Among them, TK4 contains a very high content of hydrocarbons, including Tridecane and Dodecane; TN71, KH147, and TC194 contain mainly aldehydes such as Nonanal and Hexanal. During different storage times, the contents of alcohols, monoterpenes, aromatic aldehydes, and furans increased with storage time, while the content of aliphatic aldehydes decreased with storage time. After storage, the fragrant rice samples showed a tendency for the total volatile component content to decrease, with the most pronounced reduction observed in Nonanal content.

## 1. Introduction

Rice is a one- to two-year-old herbaceous plant belonging to the *Oryza* genus of Poaceae, mainly divided into two subspecies: indica (*Oryza sativa* subsp. *indica*) and japonica (*O*. *sativa* subsp. *japonica*) [1]. Rice is one of the most important food crops in the world [2]. It can be divided into two types: ordinary rice and fragrant rice based on the presence of aroma. There are various fragrant rice varieties around the world, and they have different aroma characteristics. For example, jasmine rice from Thailand has a grass and citrus aroma [3], while Taiwanese fragrant rice (Tainung 71) has a taro aroma (Chen and Chiu, 2011) [4]. However, the aroma composition of fragrant rice with different aroma types is quite complex (Lu et al. 2023) [5].

At present, studies have proven that the key volatile compound 2-Acetyl-1-pyrroline (2-AP), which makes fragrant rice produce a special aroma, is also the main factor affecting the smell of fragrant rice [6,7]. 2-AP is produced during rice growth and can usually be detected in its leaves, stems, and grains [8]. The content of 2-AP will affect the aroma intensity of fragrant rice, and its content will decrease with storage time and temperature [9]. In addition, during the storage of fragrant rice, the key volatile compound 2-AP is significantly reduced, while lipid oxidation products are significantly increased [10].

Storage is one of the main factors affecting aroma components [11,12] and affects the composition of its aroma, and aroma is an important indicator of fragrant rice. Therefore, the purpose of this experiment was to explore the differences in the volatile components of different fragrant rice varieties and different storage times. It is hoped that this research can lead to a more in-depth study on the fragrant rice produced in Taiwan and provide an important reference for the agriculture and breeding of fragrant rice in the future.

## 2. Materials and Methods

### 2.1. Chemicals

The analytical standards used for the identification of the aroma compounds in fragrant rice included 1-Hexanol, 1-Octanol, 1-Octen-3-ol, Hexanal, Nonanal, 1-Hexanol, 1-Heptanal, Decanal, α-Pinene, 2-Acetyl-1-pyrroline, and alkanes (C_5_–C_25_), all purchased from Sigma-Aldrich (St. Louis, MO, USA). The internal standard, 2,4,6-Trimethylpyridine, was purchased from Chem Service (West Chester, PA, USA).

### 2.2. Plant Materials

A total of four varieties of fragrant rice from Taiwan were used in this study (Table 1): Taikeng No. 4 (TK4), Tainung No. 71 (TN71), Kaohsiung No. 147 (KH147), and Taichung No. 194 (TC194). Four varieties of fragrant rice were provided by the Hualien District Agricultural Research and Extension Station, Ministry of Agriculture. The identities of the plants were confirmed by Jui-Chia Lee. The samples were harvested in June 2021, and the milling degree was 85%, which was obtained after milling the brown rice. After milling, all samples were stored in hermetically sealed packages at 4 °C.

### 2.3. Method

#### 2.3.1. HS-SPME Extraction Conditions

The method used was modified from those of Dias et al. [13]:

1. Comparisons of the extraction fibers: Five different extraction fibers, 100 μm polydimethylsiloxane (PDMS), 85 μm polyacrylate (PA), 75 μm carboxen/polydimethylsiloxane (CAR/PDMS), 65 μm polydimethylsiloxane/divinylbenzene (PDMS/DVB), and 50/30 μm divinylbenzene/carboxen/polydimethylsiloxane (DVB/CAR/PDMS) (Supelco, Inc., Bellefonte, PA, USA) were used for the aroma extraction. The samples (TC194) were placed into a homogenizer (Giaretti, GL-9237, Milan, Italy). After being homogenized for 30 s, 5 g of the homogenized samples was put into a 15 mL vial (Supelco, Inc., No. 27159, Bellefonte, PA, USA) and sealed before being extracted in a water bath at 80 °C for 18 min. After sampling, each fiber was immediately inserted into the GC and GC-MS. This experiment was repeated in triplicate.
2. Comparisons of the extraction times: The above-mentioned optimal extraction fiber was used in the comparison of the extraction times. The extraction times were 14, 18, 22, 26, and 30 min to determine the optimal extraction conditions. After sampling, each fiber was immediately inserted into the GC and GC-MS.
3. Analysis of aroma components in different fragrant rice varieties: Take TK4, TN71, KH147, and TC194 fragrant rice as samples. Crush them with a homogenizer, weigh 5 g of powder, and use the above-mentioned HS-SPME under optimal experimental conditions.
4. Analysis of the aroma components of fragrant rice at different storage times: This experimental method is referred to and modified from Choi et al. [14]. TK4, TN71, KH147, and TC194 were used as samples, stored for 0, 1, 2, 4, and 6 months in sequence, and then extracted and analyzed using the above-mentioned HS-SPME optimal condition experimental method.

#### 2.3.2. Gas Chromatography (GC)

The instrument used was the model 7890A GC of the Agilent Company (Santa Clara, CA, USA), and it was matched with the company’s DB-1 (60 m × 0.25 mm × 0.25 mm) separation column; the heating condition was an initial temperature of 40 °C, maintained for 1 min, then raised to 150 °C at 5 °C/min, maintained for 1 min, and finally raised to 200 °C at 10 °C/min, which was maintained for 11 min. The detector used was the Flame Ionization Detector (FID), the injection mode was the splitless mode, the temperature of the injection port was 250 °C, and the temperature of the detector was 300 °C. The carrier gas used was nitrogen, and the gas flow rate was 1.0 mL/min.

#### 2.3.3. Gas Chromatography-Mass Spectrometry (GC-MS)

The instrument used was an Agilent Company (Santa Clara, CA, USA) model 7890B GC connected in series with a 5977A quadrupole column mass spectrometer (Mass Selective Detector, MSD; Santa Clara, CA, USA). The operating conditions and columns of the GC were the same as those of the aforementioned GC, the electron energy was 70 eV at 230 °C, and the temperature of the quadrupole column was 150 °C. The carrier gas used was changed to helium. The mass spectrum data were compared with Wiley 7N mass spectrum library.

#### 2.3.4. Retention Index (RI)

The GC-retention index of the volatile compounds in this experiment was based on a mixture of C_5_–C_25_ *n*-alkanes (Sigma-Aldrich, St. Louis, MO, USA) and the GC retention time under the same conditions as a reference, and calculated according to the method of Van Den Dool and Kratz [15].

Its retention index formula is as follows:

*I*_x_ = 100n + 100(t_x_ − t_n_)/(t_n+1_ − t_n_)

where t_n_ and t_n+1_ represent the residence time of *n*-alkanes before and after compound elution

n = carbon number of *n*-alkanes
t_x_ = residence time of the compound
x = indicates an unknown compound

#### 2.3.5. Statistical Analysis

The data were subjected to a mono-factorial variance analysis, with Duncan’s multiple range method used by a significance of differences of *p* < 0.05 (SPSS Base 12.0).

## 3. Results and Discussion

### 3.1. Comparisons of the Optimization Conditions of HS-SPME

#### 3.1.1. HS-SPME Fiber Selection

Before extracting volatile compounds, the fiber coating type will be selected according to the properties and requirements of the volatile compounds [13]. Therefore, to obtain the best extraction fibers, this experiment used five different extraction fibers to absorb fragrant rice for 18 min at a water bath temperature of 80 °C. The results showed that 100 μm PDMS and 85 μm PA were less suitable for extracting volatile compounds from fragrant rice, while 65 μm PDMS/DVB had the best extraction effect compared with 75 μm CAR/PDMS and 50/30 μm DVB/CAR/PDMS, and had a higher total volatile compound content and component quantity (Table 2).

Peng et al. [16] used three different extraction fibers (100 μm PDMS, 75 μm CAR/PDMS, and 65 μm PDMS/DVB) to analyze the volatile compounds of four fragrant rice spices, and the results showed that 65 μm PDMS/DVB fibers had the best extraction performance. According to the literature, PDMS is a nonpolar coating. If it is combined with polar coating DVB or CAR, it can not only increase the extraction effect, but also adsorb a wider range of volatile compounds with different polarities [17]. Dias et al. [13] noted that 50/30 μm DVB/CAR/PDMS fibers are more widely used to extract volatile compounds in rice because they can absorb more volatile compounds of different polarities. Although 50/30 μm DVB/CAR/PDMS fibers outperformed 65 μm PDMS/DVB fibers, 65 μm PDMS/DVB fibers were preferred because of the observed residual problem. However, when using 65 μm PDMS/DVB fibers, no residual problems were shown [18].

According to the preliminary experimental results and the discussion of the above literature, 65 μm PDMS/DVB fiber was selected as the extraction fiber for HS-SPME in this experiment. Compared with other extraction fibers, this fiber has the best extraction effect and can be used to analyze more fragrant rice aroma components.

#### 3.1.2. HS-SPME Extraction Time

**Figure 1:**
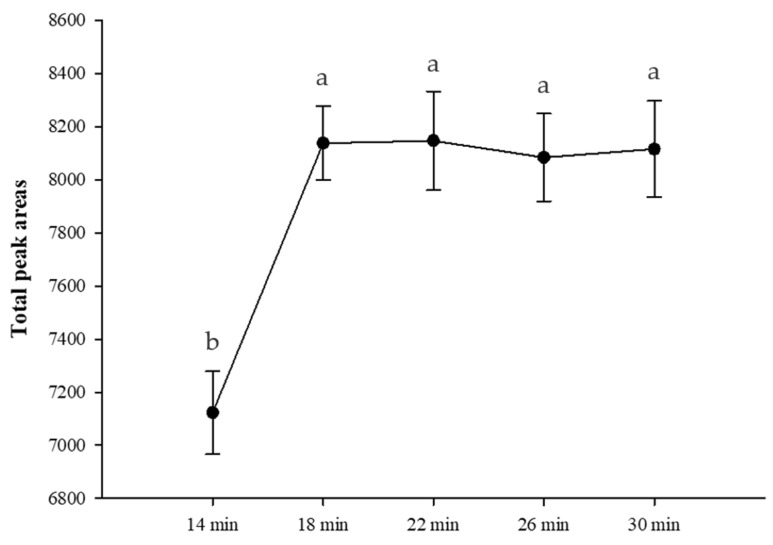
Total peak areas of fragrant rice with the 65 μm PDMS/DVB fiber at different adsorption times. Values are means ± SD of triplicates. Values with different superscripts are significantly (*p* < 0.05) different.

Based on the test results of the HS-SPME fiber, in this experiment, the 65 μm PDMS/DVB extraction fiber with the best extraction effect was used to extract fragrant rice for 14, 18, 22, 26, and 30 min in a water bath at a temperature of 80 °C. The results (Figure 1) showed that the content of total volatile compounds had a tendency to increase significantly from 14 to 18 min of extraction time, but not after 22 to 30 min, and there was no significant difference (*p* < 0.05).

Lim et al. [19] analyzed the volatile compounds of white rice and extracted the samples for 10, 20, 30, 40, and 50 min. The research results showed that as the extraction time increased, the number of peaks decreased. According to the literature, shorter extraction times are more suitable for volatile compounds, while longer extraction times are more favorable for less volatile compounds [20]. In addition, the water molecules present in the headspace are also adsorbed by the fibers during extraction, especially at higher temperatures, so longer extraction times may cause more water to accumulate on the fibers and thus reduce extraction efficiency [16]. Choi et al. [21] analyzed the volatile compounds of black rice with 50/30 μm DVB/CAR/PDMS and adsorbed them in the headspace at 80 °C for 18 min. Therefore, the experimental results of this experiment are the same as the experimental results of Choi et al. [21]. The optimal extraction condition for fragrant rice is 80 °C for 18 min, which can absorb more aroma components and meet the analysis efficiency in actual operation.

### 3.2. Volatile Compounds of Different Varieties of Fragrant Rice

**Figure 2:**
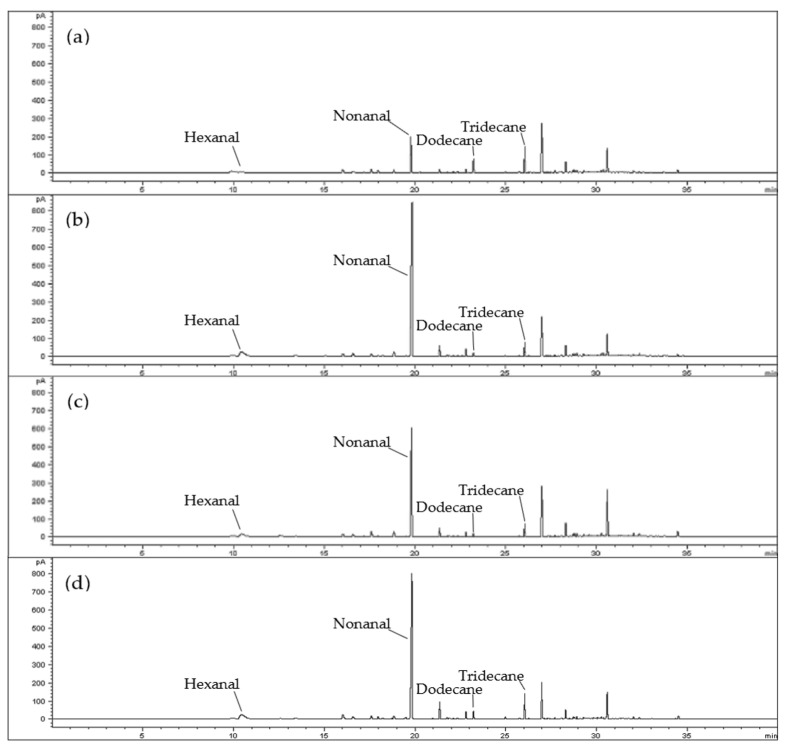
GC chromatograms of volatile compounds from TK4, TN71, KH147, and TC194 determined by HS-SPME. (**a**) TK4; (**b**) TN71; (**c**) KH147; (**d**) TC194.

In this experiment, HS-SPME was used to extract the volatile compounds of TK4, TN71, KH147, and TC194 fragrant rice, and GC-MS was used to identify and compare the composition differences (Figure 2). In Taiwan, TK4, TN71, and KH147 are generally considered to have a taro aroma, while TC194 is considered to have a popcorn aroma. The results of this experiment show that among the white rice samples of the four fragrant rice varieties, the highest content of all samples is Nonanal (1293.32–3521.70), which is one of the most important components in many rice varieties (Table 3).

The content of 2-AP was the highest in TK4 (14.36), followed by TC194 (11.32), KH147 (8.46), and TN71 (7.52). Champagne [22] pointed out that there is no single compound that can represent the characteristic aroma of fragrant rice, except for 2-AP, which could be produced by the interaction of multiple volatile compounds.

Moreover, 2-AP has the aroma of popcorn [23], and exists in some other foods, such as corn, wheat, barley, sorghum, and even taro. Therefore, 2-AP is sometimes also classified as one of the components of the taro aroma [24], while in Taiwan, some farmers believe that TK4, TN71, and KH147 have a taro aroma, and TC194 is considered to have a popcorn aroma, but in the study, all four samples contained 2-AP with little difference in content. Therefore, the speculation is only the subjective aroma perception of farmers.

**Figure 3:**
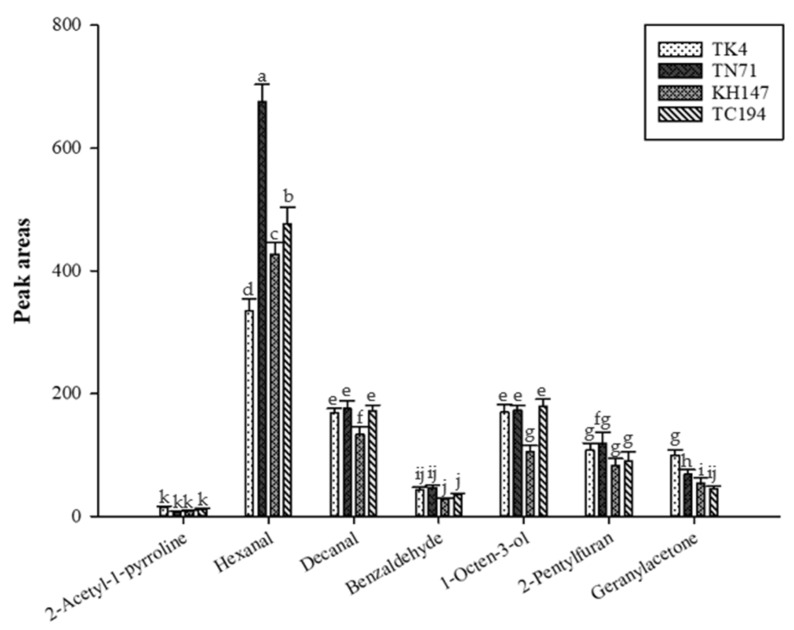
The aroma compounds from TK4, TN71, KH147, and TC194 determined by HS-SPME. Values are means ± SD of triplicates. Values with different superscripts are significantly (*p* < 0.05) different.

Zhao et al. [3] identified jasmine rice by the odor activity value (OAV) and detection frequency analysis (DFA), and indicated that Hexanal, Octanal, Nonanal, (*E*)-2-Octenal, and Decanal were the odor-active components of jasmine rice. Liu and Yang [25] used HS-SPME combined with GC-MS to investigate the influence of Yihchuan fragrant rice on volatile compounds under different milling conditions, and pointed out that Hexanal, Nonanal, Benzaldehyde, 1-Octen-3-ol, Geranylacetone, 2-Pentylfuran, and 2-AP are commonly found in the aroma profiles of fragrant rice and taro, and suggested that these volatile compounds may contribute to the taro-like flavor in fragrant rice, and the results are similar to the ingredients in this experiment (Figure 3). Hexanal and Decanal are aldehydes, which are the decomposition products of oleic acid and linolenic acid [26]. Among them, TN71 has the highest content of Hexanal, followed by TC194, KH147, and TK4. In addition, Hexanal has a grassy taste [12]. Decantal has a citrus peel-like smell [27]. Benzaldehyde has an almond fragrance [28]. In addition, 1-Octen-3-ol is a product of lipid oxidation and has an earthy and mushroomy smell [17], while 2-Pentylfuran is the reaction product of lipoxygenase (Lipoxygenase) [29], and has bean and nutty flavors [30]. Geranylacetone has a floral and fruity aroma [31], and among the four varieties, TC194 has the lowest Geranylacetone content.

In addition, TK4 contains a fairly high content of hydrocarbons, including Tridecane and Dodecane, and most of the hydrocarbons have a high threshold and have no significant impact on the overall aroma characteristics [32]. Based on the above and the experimental results, TN71, KH147, and TC194 are dominated by aldehydes, followed by hydrocarbons; TK4 is contrary to this result as it is dominated by hydrocarbons, followed by aldehydes.

### 3.3. Effects of Storage Time of Different Fragrant Rice Varieties on Aroma Components

**Figure 4:**
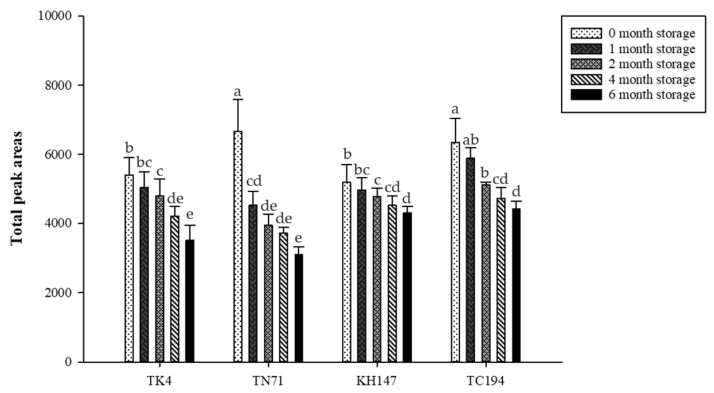
Analysis of volatile compounds in TK4, TN71, KH147, and TC194 with different storage times by HS-SPME. Values are means ± SD of triplicates. Values with different superscripts are significantly (*p* < 0.05) different.

HS-SPME was used to analyze and compare the aroma components of four fragrant rice varieties with different storage times. The results (Table 4, Table 5, Table 6, Table 7 and Table 8, Figure 4) showed that a total of 30 aroma components were identified, including 11 aliphatic hydrocarbons, 5 aliphatic alcohols, 5 aliphatic aldehydes, 1 aromatic aldehyde, 1 ketone, 1 lactone, 1 furan, 1 pyridine, 3 monoterpenes, and 1 sesquiterpene. Among them, aldehydes are the most important volatile compounds, and the content is the highest at the 0th month, but the aldehydes in the white rice of the four fragrant rice varieties all have a tendency to decrease significantly after storage. The aldehydes reached their lowest in the 6th month, among which the decline trend of Nonanal was the most obvious for this kind of compound, and TK4 decreased from 1293.32 to 261.11 in the 6th month; TN71 decreased from 3521.70 to 620.06 in the 6th month; KH147 decreased from 2218.19 to 375.99 in the 6th month; and TC194 decreased from 2968.41 to 866.89 in the 6th month. The content of hydrocarbons TK4 and TN71 was the highest at the 0th month, and there was an obvious trend of decline after storage, while that of TC194 showed a trend of increase. The aliphatic aldehydes tended to decrease after storage. According to Choi et al. [14], storage at higher temperatures can lead to an increase in the content of aldehydes (such as Nonanal), while lower temperatures can retard lipid oxidation. In addition, alcohols are considered to be the by-products of unsaturated fatty acid oxidation and formed by the further decomposition of aldehydes [9]. At the same time, alcohols are also minor volatile compounds in rice (second only to aldehydes) [33]. In this experiment, the alcohol compounds of all samples were significantly increased after storage, among which the content of 2-Ethyl-1-hexanol was the highest, and TK4 increased from 193.42 to 694.02 in the 6th month; TN71 rose from 99.02 to 323.87 in the 6th month; KH147 rose from 156.85 to 700.13 in the 6th month; and TC194 rose from 296.93 to 566.54 in the 6th month.

However, the content of ketones, lactones, furans, monoterpenes, and sesquiterpenes increased after storage, and reached the highest at the 6th month. After the storage of monoterpenes, β-Pinene was formed in the 1st month and α-Pinene was formed in the 2nd month, and its content rose to the highest in the 6th month. In the study of Bao et al. [34], monoterpene compounds such as α-pinene, β-pinene, myrcene, and limonene were indicated as biogenic volatile organic compounds emitted from rice. The results of this experiment also detected α-pinene, β-pinene, and limonene, among which the content of limonene was the highest.

In addition, with the increase in storage time, the content of 2-AP, TK4 content was the highest before storage, and the lowest after storage in the 6th month, TN71 and TC194 had the highest content in the 2nd month of storage, and gradually decreased in the 3rd month, while the content of KH147 was the highest in the 1st month, and gradually decreased in the 2nd month. Compared with the results of Tananuwong and Lertsiri [10], it was found that except for the 2-AP content of TK4, which gradually decreased with the increasing storage time, the 2-AP content of other varieties did not change significantly during different storage periods.

## 4. Conclusions

In this study, the optimal adsorption condition for the HS-SPME analysis of fragrant rice aroma compounds was extraction using a 65 μm PDMS/DVB fiber at 80 °C for 18 min. Among the TK4, TN71, KH147, and TC194 samples, the common main component is Nonanal. TN71, KH147, and TC194 are dominated by aldehydes, followed by hydrocarbons; TK4 is dominated by hydrocarbons, followed by aldehydes. Aldehydes are the most important volatile compounds during different storage periods, and their content tends to decrease significantly after storage, while the content of alcohols increases after storage. In addition, after storage, the 2-AP content in TK4 will gradually decrease.

## Figures and Tables

**Table 1:** Planting information for the fragrant rice used in this study.

Varieties	Growing Locality
Taikeng No. 4	Fuli Township, Hualien County
Tainung No. 71	Yuli Township, Hualien County
Kaohsiung No. 147	Fuli Township, Hualien County
Taichung No. 194	Fuli Township, Hualien County

**Table 2:** Comparisons of the total peak areas of total volatile compounds detected in the headspace of TC194 using different HS-SPME fibers.

HS-SPME Fibers	Peaks	Total Peak Areas
100 μm PDMS	13	3818
85 μm PA	8	2228
75 μm CAR/PDMS	16	4560
65 μm PDMS/DVB	30	8051
50/30 μm DVB/CAR/PDMS	27	4827

**Table 3:** The volatile compounds from TK4, TN71, KH147, and TC194 determined by HS-SPME.

Compound	RI ^a^	Peak Areas			
		TK4	TN71	KH147	TC194
**Aliphatic hydrocarbons**					
Undecane	1100	37.32 ± 2.05	6.77 ± 1.64	7.19 ± 1.37	8.91 ± 1.95
Dodecane	1200	519.63 ± 28.80	138.59 ± 9.73	121.52 ± 6.73	195.16 ± 4.61
1-Tridecene	1284	139.24 ± 8.05	35.69 ± 4.20	30.79 ± 1.51	34.84 ± 2.63
Tridecane	1300	775.05 ± 34.92	167.93 ± 13.34	329.48 ± 15.38	479.94 ± 18.01
3-Methyltridecane	1368	119.52 ± 8.87	30.07 ± 4.74	28.27 ± 1.26	70.04 ± 3.66
1-Tetradecene	1384	80.02 ± 6.97	94.17 ± 9.86	81.38 ± 2.38	22.50 ± 4.44
Tetradecane	1400	164.32 ± 6.16	173.83 ± 7.19	315.59 ± 15.66	194.14 ± 8.93
1-Pentadecene	1483	160.22 ± 9.95	101.81 ± 9.56	108.68 ± 8.59	42.07 ± 9.45
Pentadecane	1500	176.16 ± 9.21	105.21 ± 10.74	102.80 ± 8.01	92.69 ± 7.41
Hexadecane	1600	133.52 ± 13.62	86.43 ± 7.95	116.24 ± 9.38	88.26 ± 4.03
Heptadecane	1700	51.35 ± 1.36	38.86 ± 2.11	28.38 ± 2.01	15.34 ± 1.18
**Aliphatic alcohols**					
1-Hexanol	847	37.89 ± 2.07	62.34 ± 13.14	173.47 ± 14.68	58.31 ± 2.49
1-Hptanol	942	0.16 ± 0.01	- ^b^	-	-
1-Octanol	1040	119.47 ± 9.15	177.14 ± 11.97	163.11 ± 10.94	138.29 ± 7.27
1-Octen-3-ol	953	169.84 ± 12.09	172.93 ± 7.22	105.86 ± 9.39	179.08 ± 11.64
2-Ethyl-1-hexanol	998	193.42 ± 6.33	99.02 ± 4.61	156.85 ± 8.56	296.93 ± 14.82
**Aliphatic aldehydes**					
Hexanal	772	334.75 ± 19.30	675.27 ± 28.65	427.22 ± 19.25	476.69 ± 26.50
Heptanal	874	65.30 ± 5.97	75.82 ± 4.47	53.77 ± 3.59	78.84 ± 5.24
Nonanal	1070	1293.32 ± 41.87	3521.70 ± 49.41	2218.19 ± 79.14	2968.41 ± 53.73
(*E*)-2-Nonenal	1123	182.64 ± 6.55	302.59 ± 15.96	133.65 ± 8.43	409.08 ± 11.81
Decanal	1174	168.31 ± 7.31	175.88 ± 12.56	134.01 ± 12.40	171.60 ± 9.90
**Aromatic aldehyde**					
Benzaldehyde	923	43.52 ± 4.40	47.17 ± 3.49	28.68 ± 1.53	36.48 ± 1.32
**Ketone**					
2-Heptanone	860	0.15 ± 0.02	0.29 ± 0.01	-	-
**Lactone**					
Geranylacetone	1415	99.71 ± 7.82	68.59 ± 7.56	54.60 ± 8.24	45.35 ± 3.56
**Furan**					
2-Pentylfuran	970	107.49 ± 12.17	118.72 ± 18.70	82.13 ± 11.84	90.82 ± 13.96
**Pyridine**					
2-Acetyl-1-pyrroline	886	14.36 ± 2.59	7.52 ± 0.66	8.46 ± 1.69	11.32 ± 1.79
**Monoterpene**					
Limonene	1011	51.17 ± 4.01	18.40 ± 2.57	30.77 ± 1.81	45.66 ± 5.75
**Sesquiterpene**					
Longifolene	1406	113.03 ± 9.15	120.29 ± 10.91	108.04 ± 8.13	74.41 ± 2.00
**Total**		5350.84 ± 496.58	6623.03 ± 633.52	5149.13 ± 511.44	6325.16 ± 679.85

^a^ Rentention indices, using paraffin (C_5_–C_25_) as references; ^b^ undectable.

**Table 4:** Analysis of volatile compounds in TK4 with different storage times by HS-SPME.

Compound	RI ^a^	Peak Areas				
		0 M ^b^	1 M	2 M	4 M	6 M
**Aliphatic hydrocarbons**						
Undecane	1100	37.32 ± 2.05	28.12 ± 3.01	20.59 ± 2.16	29.41 ± 2.32	31.71 ± 5.72
Dodecane	1200	519.63 ± 28.80	203.69 ± 12.68	189.66 ± 16.51	110.02 ± 13.60	80.97 ± 9.28
1-Tridecene	1284	139.24 ± 8.05	83.91 ± 3.41	78.93 ± 2.82	84.58 ± 2.61	91.34 ± 4.35
Tridecane	1300	775.05 ± 34.92	490.22 ± 30.49	379.04 ± 25.35	273.32 ± 16.56	133.97 ± 24.78
3-Methyltridecane	1368	119.52 ± 8.87	106.97 ± 2.87	93.46 ± 5.87	70.22 ± 6.56	62.06 ± 8.16
1-Tetradecene	1384	80.02 ± 6.97	86.64 ± 8.20	85.64 ± 2.40	85.64 ± 2.12	85.64 ± 4.61
Tetradecane	1400	164.32 ± 6.16	157.12 ± 5.24	142.06 ± 12.97	137.97 ± 9.37	129.24 ± 2.34
1-Pentadecene	1483	160.22 ± 9.95	169.65 ± 4.76	130.97 ± 13.86	124.54 ± 14.28	106.32 ± 12.30
Pentadecane	1500	176.16 ± 9.21	167.12 ± 12.83	155.63 ± 4.25	140.11 ± 5.20	117.36 ± 7.46
Hexadecane	1600	133.52 ± 13.62	140.38 ± 4.28	135.29 ± 8.05	140.43 ± 14.65	134.65 ± 5.63
Heptadecane	1700	51.35 ± 1.36	46.22 ± 2.78	51.68 ± 0.96	45.84 ± 3.12	45.84 ± 1.49
**Aliphatic alcohols**						
1-Hexanol	847	37.89 ± 2.07	33.25 ± 1.42	30.78 ± 2.77	25.18 ± 2.81	24.46 ± 1.60
1-Hptanol	942	0.16 ± 0.01	5.16 ± 0.84	9.16 ± 1.08	15.79 ± 1.45	20.20 ± 2.73
1-Octanol	1040	119.47 ± 9.15	125.02 ± 9.66	124.47 ± 8.21	120.62 ± 11.17	121.76 ± 6.54
1-Octen-3-ol	953	169.84 ± 12.09	123.49 ± 15.39	115.06 ± 7.43	109.78 ± 10.38	97.09 ± 13.63
2-Ethyl-1-hexanol	998	193.42 ± 6.33	639.25 ± 18.16	658.58 ± 17.51	670.33 ± 28.44	694.02 ± 13.89
**Aliphatic aldehydes**						
Hexanal	772	334.75 ± 19.30	282.68 ±29.66	238.99 ± 16.68	192.62 ± 12.05	168.51 ± 9.21
Heptanal	874	65.30 ± 5.97	60.86 ± 5.17	59.41 ± 7.10	57.76 ± 2.83	53.51 ± 1.33
Nonanal	1070	1293.32 ± 41.87	1160.54 ± 51.26	1110.54 ± 22.63	751.97 ± 26.28	261.11 ± 25.45
(*E*)-2-Nonenal	1123	182.60 ± 6.55	165.62 ± 14.40	144.96 ± 4.54	109.64 ± 1.70	83.13 ± 8.40
Decanal	1174	168.31 ± 7.31	169.19 ± 9.83	160.37 ± 7.78	123.58 ± 11.78	103.67 ± 17.15
**Aromatic aldehyde**						
Benzaldehyde	923	43.52 ± 4.40	31.19 ± 5.32	35.52 ± 3.40	61.59 ± 2.62	73.30 ± 14.76
**Ketone**						
2-Heptanone	860	0.15 ± 0.02	16.15 ± 2.93	26.15 ± 4.14	43.10 ± 3.94	45.08 ± 2.52
**Lactone**						
Geranylacetone	1415	99.71 ± 7.82	143.71 ± 9.73	176.25 ± 12.24	181.37 ±7.82	198.75 ± 13.62
**Furan**						
2-Pentylfuran	970	107.49 ± 12.17	135.85 ± 8.67	139.17 ± 14.58	140.95 ± 12.77	143.16 ± 13.11
**Pyridine**						
2-Acetyl-1-pyrroline	886	14.36 ± 2.59	11.61 ± 1.18	11.50 ± 1.09	10.17 ± 1.26	9.08 ± 0.76
**Monoterpenes**						
α-Pinene	925	- ^c^	0.16 ± 0.02	2.96 ± 0.94	7.70 ± 1.22	13.81 ± 1.08
β-Pinene	965	-	6.38 ± 1.07	19.38 ± 5.30	59.92 ± 6.17	73.22 ± 9.87
Limonene	1011	51.17 ± 4.01	74.13 ± 7.38	80.23 ± 10.68	84.07 ± 7.26	91.20 ± 11.56
**Sesquiterpene**						
Longifolene	1406	113.03 ± 9.15	109.11 ± 9.45	109.41 ± 12.82	111.14 ± 10.04	102.65 ± 9.09
**Total**		5350.84 ± 496.58	4973.39 ± 453.64	4715.84 ± 490.11	4119.33 ± 276.93	3396.81 ± 431.77

^a^ Rentention indices, using paraffin (C_5_–C_25_) as references; ^b^ storage time (month); ^c^ undectable.

**Table 5:** Analysis of volatile compounds in TN71 with different storage times by HS-SPME.

Compound	RI ^a^	Peak Areas				
		0 M ^b^	1 M	2 M	4 M	6 M
**Aliphatic hydrocarbons**						
Undecane	1100	6.77 ± 1.64	6.27 ± 3.59	8.57 ± 1.61	10.54 ± 0.63	9.47 ± 2.37
Dodecane	1200	138.59 ± 9.73	133.62 ± 13.23	117.60 ± 3.23	105.15 ± 5.57	90.56 ± 9.82
1-Tridecene	1284	35.69 ± 4.20	35.77 ± 6.22	40.77 ± 1.92	42.51 ± 1.64	42.93 ± 3.65
Tridecane	1300	167.93 ± 13.34	150.52 ± 8.29	140.52 ± 9.70	139.97 ± 6.36	125.12 ± 15.07
3-Methyltridecane	1368	30.07 ± 4.74	35.31 ± 6.09	40.31 ± 1.61	48.32 ± 1.26	50.82 ± 2.02
1-Tetradecene	1384	94.17 ± 9.86	73.07 ± 9.02	66.07 ± 2.09	59.97 ± 3.85	54.85 ± 0.46
Tetradecane	1400	173.83 ± 7.19	130.69 ± 6.19	120.69 ± 14.67	117.11 ± 4.38	114.25 ± 9.95
1-Pentadecene	1483	101.81 ± 9.56	118.72 ± 2.47	128.72 ± 9.80	123.97 ± 7.85	121.29 ± 7.52
Pentadecane	1500	105.21 ± 10.74	95.11 ± 4.73	94.11 ± 5.23	90.65 ± 8.25	88.76 ± 8.43
Hexadecane	1600	86.43 ± 7.95	79.29 ± 9.37	69.29 ± 9.40	68.91 ± 5.78	60.39 ± 6.94
Heptadecane	1700	38.86 ± 2.11	42.88 ± 1.39	44.89 ± 1.16	44.53 ± 3.71	48.44 ± 2.65
**Aliphatic alcohols**						
1-Hexanol	847	62.34 ± 13.14	60.09 ± 9.76	68.09 ± 8.61	65.69 ± 4.66	67.13 ± 3.81
1-Hptanol	942	- ^c^	-	3.94 ± 1.03	10.72 ± 0.35	13.27 ± 1.69
1-Octanol	1040	177.14 ± 11.97	113.75 ± 9.49	99.75 ± 7.92	85.61 ± 6.43	78.57 ± 6.08
1-Octen-3-ol	953	172.93 ± 7.22	159.87 ± 8.62	116.87 ± 6.48	110.33 ± 10.04	101.59 ± 9.21
2-Ethyl-1-hexanol	998	99.02 ± 4.61	183.72 ± 7.87	253.72 ± 15.47	280.77 ± 11.43	323.87 ± 7.65
**Aliphatic aldehydes**						
Hexanal	772	675.27 ± 28.65	405.06 ± 14.57	370.06 ± 14.52	326.72 ± 24.57	303.20 ± 7.58
Heptanal	874	75.82 ± 4.47	64.86 ± 3.99	50.86 ± 2.83	46.95 ± 8.13	40.83 ± 2.25
Nonanal	1070	3521.70 ± 49.41	1952.17 ± 28.75	1432.17 ± 42.63	1125.25 ± 27.22	620.06 ± 16.42
(*E*)-2-Nonenal	1123	302.59 ± 15.96	159.91 ± 5.24	103.91 ± 3.00	93.31 ± 2.87	58.25 ± 5.41
Decanal	1174	175.88 ± 12.56	153.41 ± 8.21	139.41 ± 5.21	119.13 ± 7.14	111.26 ± 9.44
**Aromatic aldehyde**						
Benzaldehyde	923	47.17 ± 3.49	49.15 ± 1.78	50.15 ± 3.55	54.87 ± 1.81	56.47 ± 1.55
**Ketone**						
2-Heptanone	860	0.29 ± 0.01	7.65 ± 0.15	11.30 ± 1.35	18.54 ± 0.21	34.22 ± 2.78
**Lactone**						
Geranylacetone	1415	68.59 ± 7.56	73.55 ± 4.07	93.55 ± 8.71	106.98 ± 3.10	121.16 ± 5.08
**Furan**						
2-Pentylfuran	970	118.72 ± 18.70	123.46 ± 6.72	136.46 ± 8.32	140.25 ± 3.95	143.06 ± 4.78
**Pyridine**						
2-Acetyl-1-pyrroline	886	7.52 ± 0.66	8.48 ± 0.40	10.48 ± 0.31	9.10 ± 0.47	8.52 ± 0.81
**Monoterpenes**						
α-Pinene	925	-	-	0.19 ± 0.02	5.19 ± 0.17	4.82 ± 0.11
β-Pinene	965	-	0.20 ± 0.02	12.12 ± 1.67	20.78 ± 1.71	25.03 ± 1.52
Limonene	1011	18.40 ± 2.57	49.05 ± 7.69	54.05 ± 6.70	62.25 ± 4.83	85.07 ± 5.40
**Sesquiterpene**						
Longifolene	1406	120.29 ± 10.91	47.62 ± 7.88	44.62 ± 5.82	165.15 ± 9.93	56.53 ± 4.83
**Total**		6623.03 ± 633.52	4513.25 ± 398.91	3923.24 ± 315.85	3699.22 ± 145.39	3059.79 ± 211.27

^a^ Rentention indices, using paraffin (C_5_–C_25_) as references; ^b^ storage time (month); ^c^ undectable.

**Table 6:** Analysis of volatile compounds in KH147 with different storage times by HS-SPME.

Compound	RI ^a^	Peak Areas				
		0 M ^b^	1 M	2 M	4 M	6 M
**Aliphatic hydrocarbons**						
Undecane	1100	7.19 ± 1.37	29.12 ± 4.76	45.26 ± 3.47	53.12 ± 4.66	80.26 ± 3.10
Dodecane	1200	121.52 ± 6.73	94.06 ± 4.44	80.81 ± 9.04	74.06 ± 1.61	70.81 ± 6.09
1-Tridecene	1284	30.79 ± 1.51	64.83 ± 13.26	70.59 ± 7.76	72.83 ± 7.61	74.59 ± 7.11
Tridecane	1300	329.48 ± 15.38	319.49 ± 18.45	304.39 ± 9.70	290.49 ± 8.73	248.39 ± 18.55
3-Methyltridecane	1368	28.27 ± 1.26	55.50 ± 6.10	90.61 ± 4.00	120.50 ± 9.23	135.61 ± 7.27
1-Tetradecene	1384	81.38 ± 2.38	66.30 ± 11.69	60.77 ± 8.15	54.49 ± 4.74	50.77 ± 2.02
Tetradecane	1400	315.59 ± 15.66	295.89 ± 12.10	185.62 ± 8.21	179.89 ± 12.55	125.62 ± 4.43
1-Pentadecene	1483	108.68 ± 8.59	95.47 ± 1.09	87.79 ± 3.91	85.47 ± 8.97	84.79 ± 3.21
Pentadecane	1500	102.80 ± 8.01	109.80 ± 12.28	170.76 ± 6.62	182.80 ± 5.61	207.76 ± 5.50
Hexadecane	1600	116.24 ± 9.38	123.99 ± 7.12	130.77 ± 8.21	136.99 ± 74.26	137.77 ± 9.50
Heptadecane	1700	28.38 ± 2.01	28.84 ± 2.21	33.17 ± 1.82	33.89 ± 3.17	43.17 ± 0.70
**Aliphatic alcohols**						
1-Hexanol	847	173.47 ± 14.68	180.78 ± 12.48	187.61 ± 16.24	192.78 ± 15.70	192.61 ± 7.66
1-Hptanol	942	- ^c^	0.20 ± 0.03	6.68 ± 0.96	28.06 ± 1.30	46.68 ± 1.97
1-Octanol	1040	163.11 ± 10.94	155.98 ± 14.82	148.98 ± 6.25	125.98 ± 6.03	115.98 ± 4.53
1-Octen-3-ol	953	105.86 ± 9.39	226.89 ± 8.12	230.95 ± 8.45	246.89 ± 9.26	230.95 ± 8.45
2-Ethyl-1-hexanol	998	156.85 ± 8.56	283.00 ± 15.94	570.13 ± 9.81	693.00 ± 7.33	700.13 ± 11.12
**Aliphatic aldehydes**						
Hexanal	772	427.22 ± 19.25	345.03 ± 9.07	308.81 ± 15.51	225.03 ± 11.22	178.81 ± 5.75
Heptanal	874	53.77 ± 3.59	62.48 ± 4.11	81.08 ± 9.23	102.48 ± 8.18	121.08 ± 6.14
Nonanal	1070	2218.19 ± 79.14	1787.15 ± 75.49	1259.99 ± 39.12	727.15 ± 51.81	375.99 ± 47.95
(*E*)-2-Nonenal	1123	133.65 ± 8.43	93.57 ± 9.98	79.02 ± 5.60	75.57 ± 4.52	57.02 ± 5.37
Decanal	1174	134.01 ± 12.40	137.70 ± 10.20	140.02 ± 16.86	152.70 ± 14.95	166.02 ± 5.98
**Aromatic aldehyde**						
Benzaldehyde	923	28.68 ± 1.53	40.24 ± 7.21	47.61 ± 1.44	60.24 ± 4.14	70.61 ± 4.79
**Ketone**						
2-Heptanone	860	-	0.14 ± 0.03	7.55 ± 1.15	20.10 ± 1.73	13.55 ± 0.81
**Lactone**						
Geranylacetone	1415	54.60 ± 8.24	70.65 ± 13.99	90.18 ± 7.54	101.65 ± 11.06	108.85 ± 9.54
**Furan**						
2-Pentylfuran	970	82.13 ± 11.84	90.94 ± 7.02	98.85 ± 1.88	130.94 ± 12.26	160.18 ± 7.07
**Pyridine**						
2-Acetyl-1-pyrroline	886	8.46 ± 1.69	13.27 ± 1.46	11.86 ± 2.11	10.27 ± 0.34	9.86 ± 1.01
**Monoterpenes**						
α-Pinene	925	-	-	0.20 ± 0.05	4.63 ± 0.39	3.58 ± 0.60
β-Pinene	965	-	0.12 ± 0.04	9.80 ± 9.51	42.78 ± 5.35	49.80 ± 3.84
Limonene	1011	30.77 ± 1.81	46.59 ± 6.37	50.91 ± 9.36	60.59 ± 9.47	107.91 ± 17.54
**Sesquiterpene**						
Longifolene	1406	108.04 ± 8.13	109.76 ± 7.00	115.44 ± 11.11	129.76 ± 18.41	138.44 ± 13.18
**Total**		5149.13 ± 511.44	4927.78 ± 339.22	4706.21 ± 221.94	4415.13 ± 262.48	4107.59 ± 182.06

^a^ Rentention indices, using paraffin (C_5_–C_25_) as references; ^b^ storage time (month); ^c^ undectable.

**Table 7:** Analysis of volatile compounds in TC194 with different storage times by HS-SPME.

Compound	RI ^a^	Peak Areas				
		0 M ^b^	1 M	2 M	4 M	6 M
**Aliphatic hydrocarbons**						
Undecane	1100	8.91 ± 1.95	9.93 ± 2.87	15.93 ± 1.60	15.94 ± 1.70	16.15 ± 3.42
Dodecane	1200	195.16 ± 4.61	186.98 ± 7.29	156.98 ± 10.23	135.55 ± 2.66	126.26 ± 6.24
1-Tridecene	1284	34.84 ± 2.63	51.49 ± 10.02	73.54 ± 8.32	80.24 ± 2.57	81.43 ± 4.04
Tridecane	1300	479.94 ± 18.01	320.35 ± 15.21	212.35 ± 23.87	190.85 ± 17.90	188.40 ± 26.72
3-Methyltridecane	1368	70.04 ± 3.66	86.65 ± 13.74	96.45 ± 13.34	106.59 ± 5.05	130.54 ± 7.83
1-Tetradecene	1384	22.50 ± 4.44	68.94 ± 7.59	82.94 ± 22.83	92.31 ± 9.14	112.28 ± 6.68
Tetradecane	1400	194.14 ± 8.93	186.99 ± 10.17	166.90 ± 18.27	143.16 ± 23.90	128.79 ± 12.90
1-Pentadecene	1483	42.07 ± 9.45	51.70 ± 9.59	61.70 ± 8.09	89.94 ± 7.68	111.60 ± 16.49
Pentadecane	1500	92.69 ± 7.41	118.65 ± 14.90	188.65 ± 12.09	209.47 ± 19.58	230.05 ± 23.63
Hexadecane	1600	88.26 ± 4.03	149.73 ± 11.41	173.77 ± 11.41	208.62 ± 7.48	218.31 ± 6.86
Heptadecane	1700	15.34 ± 1.18	55.96 ± 9.64	74.96 ± 4.15	79.23 ± 12.57	85.76 ± 3.08
**Aliphatic alcohols**						
1-Hexanol	847	58.31 ± 2.49	68.78 ± 1.32	72.64 ± 6.50	75.82 ± 3.78	80.23 ± 9.12
1-Hptanol	942	- ^c^	-	4.26 ± 0.71	18.37 ± 1.55	19.76 ± 3.92
1-Octanol	1040	138.29 ± 7.27	114.16 ± 11.91	108.16 ± 6.99	84.23 ± 7.97	79.00 ± 6.90
1-Octen-3-ol	953	179.08 ± 11.64	166.66 ± 15.15	154.66 ± 12.18	147.75 ± 8.26	126.63 ± 14.42
2-Ethyl-1-hexanol	998	296.93 ± 14.82	432.86 ± 16.93	472.86 ± 16.31	526.94 ± 20.54	566.54 ± 11.33
**Aliphatic aldehydes**						
Hexanal	772	476.69 ± 26.50	404.28 ± 13.06	276.28 ± 23.52	206.54 ± 21.40	194.70 ± 9.94
Heptanal	874	78.84 ± 5.24	83.79 ± 6.34	85.79 ± 8.34	90.97 ± 7.82	96.06 ± 4.71
Nonanal	1070	2968.41 ± 53.73	2445.27 ± 35.44	1777.27 ± 32.73	1309.70 ± 24.04	866.89 ± 23.48
(*E*)-2-Nonenal	1123	409.08 ± 11.81	280.25 ± 9.31	201.25 ± 15.73	149.94 ± 8.04	94.94 ± 5.71
Decanal	1174	171.60 ± 9.90	178.80 ± 5.80	186.89 ± 12.33	193.14 ± 7.00	224.87 ± 7.39
**Aromatic aldehyde**						
Benzaldehyde	923	36.48 ± 1.32	44.13 ± 2.39	49.13 ± 5.49	61.48 ± 2.35	71.78 ± 2.83
**Ketone**						
2-Heptanone	860	-	0.23 ± 0.05	6.65 ± 0.71	10.76 ± 0.70	13.42 ± 2.40
**Lactone**						
Geranylacetone	1415	45.35 ± 3.56	54.16 ± 4.93	59.16 ± 5.03	63.88 ± 3.03	78.85 ± 5.15
**Furan**						
2-Pentylfuran	970	90.82 ± 13.96	91.74 ± 7.71	92.74 ± 6.46	97.93 ± 5.04	101.00 ± 26.71
**Pyridine**						
2-Acetyl-1-pyrroline	886	11.32 ± 1.79	12.62 ± 1.36	13.62 ± 0.83	12.98 ± 1.62	11.79 ± 1.55
**Monoterpenes**						
α-Pinene	925	-	-	0.18 ± 0.01	5.10 ± 0.37	5.34 ± 0.25
β-Pinene	965	-	4.79 ± 0.65	16.23 ± 1.41	36.71 ± 1.25	40.10 ± 2.17
Limonene	1011	45.66 ± 5.75	95.17 ± 7.01	96.17 ± 3.72	100.47 ± 4.63	108.06 ± 13.79
**Sesquiterpene**						
Longifolene	1406	74.41 ± 2.00	81.16 ± 4.30	93.16 ± 9.34	101.85 ± 5.01	128.36 ± 11.80
**Total**		6325.16 ± 679.85	5846.22 ± 302.00	5071.27 ± 69.64	4646.46 ± 320.29	4337.89 ± 224.74

^a^ Rentention indices, using paraffin (C_5_–C_25_) as references; ^b^ storage time (month); ^c^ undectable.

**Table 8:** Concentration of functional groups in TK4, TN71, KH147, and TC194 with different storage times.

Compound	Peak Areas									
	TK4					TN71				
	0 M ^a^	1 M	2 M	4 M	6 M	0 M	1 M	2 M	4 M	6 M
**Aliphatic hydrocarbons**	2356.35	1680.04	1462.95	1242.08	1019.10	979.36	901.25	871.54	851.63	806.88
**Aliphatic alcohols**	520.78	926.17	938.05	941.70	957.53	511.43	517.43	542.37	553.12	584.43
**Aliphatic aldehydes**	2044.28	1838.89	1714.27	1235.57	669.93	4751.26	2735.41	2096.41	1711.36	1133.60
**Aromatic aldehyde**	43.52	31.19	35.52	61.59	73.30	47.17	49.15	50.15	54.87	56.47
**Ketone**	0.15	16.15	26.15	43.10	45.08	0.29	7.65	11.30	18.54	34.22
**Lactone**	99.71	143.71	176.25	181.37	198.75	68.59	73.55	93.55	106.98	121.16
**Furan**	107.49	135.85	139.17	140.95	143.16	118.72	123.46	136.46	140.25	143.06
**Pyridine**	14.36	11.61	11.50	10.17	9.08	7.52	8.48	10.48	9.10	8.52
**Monoterpenes**	51.17	80.67	102.57	151.69	178.23	18.40	49.25	66.36	88.22	114.92
**Sesquiterpene**	113.03	109.11	109.41	111.14	102.65	120.29	47.62	44.62	165.15	56.53
**Total**	5350.84	4973.39	4715.84	4119.33	3396.81	6623.03	4513.25	3923.24	3699.22	3059.79
**Compound**	**KH147**					**TC194**				
**Aliphatic hydrocarbons**	1270.32	1283.29	1260.54	1284.53	1259.54	1243.89	1287.37	1304.17	1351.90	1429.57
**Aliphatic alcohols**	599.29	846.85	1144.35	1286.71	1324.35	672.61	782.46	812.58	853.11	872.16
**Aliphatic aldehydes**	2966.84	2425.93	1868.92	1282.93	898.92	4104.62	3392.39	2518.48	1950.29	1477.46
**Aromatic aldehyde**	28.68	40.24	47.61	60.24	70.61	36.48	44.13	49.13	61.48	71.78
**Ketone**	- ^b^	0.14	7.55	20.10	13.55	-	0.23	6.65	10.76	13.42
**Lactone**	54.60	70.65	90.18	101.65	108.85	45.35	54.16	59.16	63.88	78.85
**Furan**	82.13	90.94	98.85	130.94	160.18	90.82	91.74	92.74	97.93	101.00
**Pyridine**	8.46	13.27	11.86	10.27	9.86	11.32	12.62	13.62	12.98	11.79
**Monoterpenes**	30.77	46.71	60.91	108.00	161.29	45.66	99.96	112.58	142.28	153.50
**Sesquiterpene**	108.04	109.76	115.44	129.76	138.44	74.41	81.16	93.16	101.85	128.36
**Total**	5149.13	4927.78	4706.21	4415.13	4107.59	6325.16	5846.22	5071.27	4646.46	4337.89

^a^ Storage time (month); ^b^ undectable.

## Data Availability

Data sharing not applicable. No new data were created or analyzed in this study. Data sharing is not applicable to this article.
